# Prevalence of placenta previa among deliveries: An update systematic review and meta-analysis after the introduction of the two-child policy in Mainland China

**DOI:** 10.7189/jogh.14.04108

**Published:** 2024-06-14

**Authors:** Dazhi Fan, Yushi Liu, Pengzhen Hu, Dongxin Lin, Jiaming Rao, Li Sun, Wen Wang, Linlin Wu, Li Liu, Yubo Ma, Zhengping Liu, Xiaoling Guo

**Affiliations:** 1 Foshan Fetal Medicine Research Institute, Foshan Women and Children Hospital, Foshan, Guangdong, China; 2 Department of Obstetrics, Foshan Women and Children Hospital, Foshan, Guangdong, China; 3 The Second School of Clinical Medicine, Southern Medical University, Guangzhou, Guangdong, China; 4 Department of Library, Foshan Women and Children Hospital, Foshan, Guangdong, China; 5 Department of Library, First Affiliated Hospital, College of Medicine, Zhejiang University, Hangzhou, Zhejiang, China; 6 Department of Epidemiology and Biostatistics, School of Public Health, Anhui Medical University, Hefei, Anhui, China

## Abstract

**Background:**

As birth policy can affect maternal and infant health, we sought to identify whether and how the introduction of the two-child policy might have affected the prevalence of placenta previa in pregnant women in mainland China.

**Methods:**

In this update meta-analysis and systematic review, we searched PubMed, Web of Science, the Cochrane Library, Weipu, Wanfang, and the China National Knowledge Infrastructure (CNKI) databases for studies evaluating the prevalence of placenta previa in China published between the inception of each database and March 2024, with no restrictions. Two investigators independently extracted the data from each included study. We then combined the prevalence of placenta previa using random-effects models.

**Results:**

We included 128 studies in our analysis, 48 more than in our previous review. The prevalence of placenta previa among Chinese pregnant women was 1.44% (95% confidence interval (CI) = 1.32, 1.56). After the implementation of the two-child policy, the prevalence increased significantly, from 1.25% (95% CI = 1.16, 1.34) to 4.12% (95% CI = 3.33, 4.91).

**Conclusions:**

The prevalence of placenta previa increased significantly from the one-child policy period to the two-child policy period among mainland Chinese pregnant women, with varying trends across regions. This change requires the attention of health officials and timely adjustment of resource allocation policies.

**Registration:**

PROSPERO: CRD42021262309.

Placenta previa is a potentially severe obstetric complication where the placenta overlying the endocervical os [1,2]. According to a systematic review from 2013, its prevalence varies widely between countries and regions, ranging between 12.2‰ in Asia, 3.6‰ in Europe, 2.9‰ in North America, and 2.7‰ in sub-Saharan Africa [3]. Moreover, in our 2016 systematic review and meta-analysis of 80 studies that collected data between 1965 and 2015, we observed that the prevalence of placenta previa was 1.24% in mainland China, with likewise unequal geographic distribution, ranging from 2.90% in Hainan to 0.54% in Liaoning [4].

Many maternal and foetal factors are associated with placenta previa, including maternal behavioural and sociodemographic characteristics, endometrial disease or injury, and delayed development of the fertilised egg trophoblast [5–7]. The prevalence of placenta previa has increased rapidly worldwide in recent years, likely due to increases in the rates of advanced maternal age and the number of caesarean sections [8,9].

Some studies have shown that the change of birth policy is associated with increases in the proportion of advanced maternal age and assisted reproductive pregnancy, which in turn might exacerbate maternal and infant health risks [10–13]. In October 2015, China introduced the two-child policy [14], which over time led to increases in the number of women of advanced maternal age; women who had undergone caesarean sections or had uterine scars; women who used assisted reproductive technology, and women who had multiple pregnancies [15–18]. Previous studies have shown that these factors might increase the risk of adverse pregnancy complications, including placenta previa [19–23].

The temporal changes and trends of placenta previa in Chinese pregnant women need to be better understood, especially after the implementation of the two-child policy. Such data could inform current and future health care planning, as well as help identify and forecast unmet medical needs in this context. Moreover, by modifying their obstetric service strategies within the framework of the new two-child policy, hospitals could decrease adverse pregnancy outcomes and improve the quality of perinatal health care.

However, there is currently a lack of data on and no systematic analysis of the prevalence of placenta previa among Chinese pregnant women before and after the implementation of the universal two-child policy. Our study aimed to identify the prevalence and main risk factors for placenta previa following the implementation of the universal two-child policy by updating our previous meta-analysis and systematic review. We hypothesised that the prevalence of placenta previa would show an increasing trend and that its geographical distribution would change with the introduction of the two-child policy.

## METHODS

We registered the original protocol of our previous study within PROSPERO (CRD42021262309) and reported our findings per the MOOSE and PRISMA guidelines [24,25].

### Search strategy

For this updated review, we searched Web of Science, the Cochrane Library, PubMed, Weipu, Wanfang, and the China National Knowledge Infrastructure (CNKI) databases for studies that evaluated placenta previa prevalence and were published between inception and March 2024. We set no restrictions on language. With the help of two biomedical librarians (LS and LL), we designed our search around the following keywords: ‘placenta previa’ AND ‘Chinese’ AND ‘prevalence’ (File S1 in the **Online Supplementary Document**). Following deduplication, DF and DL screened the retrieved records to identify studies meeting our inclusion criteria.

### Eligibility criteria

As in our previous review [4], we included observational studies (cross-sectional, cohort studies, and case-control) on placenta previa among pregnant women meeting the clinical diagnostic criteria [26,27] conducted in mainland China, providing they reported the prevalence or incidence of placenta previa. We excluded meeting abstracts; case reports and case series; poster presentations; and articles we could not access or that had incomplete data.

### Data extraction

At least two of seven investigators (DF, LL, YL, JR, LS, PH, and WW) independently extracted the following data for each study: author, design, year of publication, sample sizes, prevalence estimates, and associated risk factors. An independent investigator (JR) verified all the extracted data. We resolved discrepancies during data extraction by discussion or adjudication by a third investigator (XG) where needed.

### Methodological quality assessment

In line with our previous reviews and similar studies [4,28–30], two investigators (DF and DL) independently assessed the risk of bias in each included study using the STROBE checklist [31] (Table S2 in the **Online Supplementary Document**). This assessment was based on five modules: sample population, sample size, participation rate, outcome assessment, and analytical methods. Each module was scored as 0 for high risk and unclear, 1 for moderate risk, and 2 for low risk, with the sum of the scores for all five modules indicating the overall bias risk of each study. We resolve discrepancies during the risk of bias assessment by discussion or adjudication by a third investigator (JR) where needed.

### Statistical analysis

The primary outcome was the prevalence of placenta previa among Chinese pregnant women. We collected raw data from every study to recalculate prevalence and their associated standard errors, using the mid-year as the reference for point prevalence when multiple years were included in the study period. We then used the *I^2^* statistic to assess the heterogeneity between included studies: *I^2^* values of 0–50%, 50–75%, and >75% were considered as low, moderate, and high, respectively. We used a random effects model for cases where *I^2^* was ≥50%; otherwise, we used a fixed effects model [32–35].

Due to the anticipated heterogeneity, we used random effects models to calculate the overall and subgroup pooled prevalence estimates [30,36]. Meanwhile, we conducted subgroup and meta-regression (univariate and multivariable) analyses to explore potential sources of heterogeneity. Based on the studies included in our previous review and other similar prevalence meta-analyses [3,4,17,18], the potential variables were geographical study region, survey year, quality score, maternal age, and hospital level. Lastly, we used funnel plots and Egger’s test to assess small-study effects. We performed all analyses in Stata, version 12.0 (StataCorp LLC, College Station, TX, USA). Statistical significance was set at *P* < 0.05.

To investigate the impact of the two-child policy, we analysed the data on the prevalence of placenta previa after 2016, i.e. after the year of the implementation of the two-child policy. To determine the geographical differences in the prevalence of placenta previa, we generated a map of pooled prevalence in each province through ArcGIS, version 10 (Environmental Systems Research Institute, Redlands, California) system [37] after calculating the pooled prevalence for each province through a meta-analysis.

## RESULTS

### Literature search

**Figure 1:**
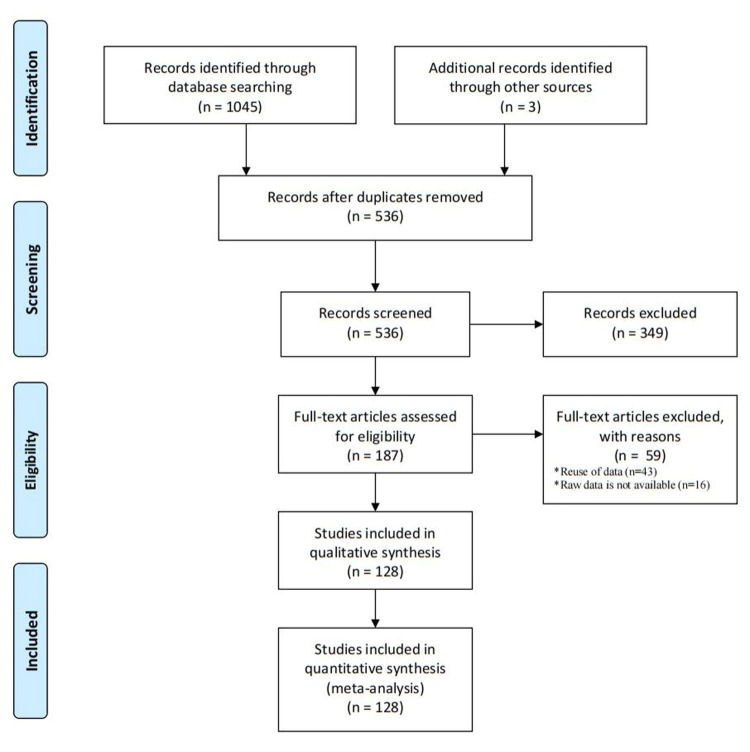
Flow diagram of the selection process.

We identified 536 non-duplicate records in the database search; 349 were ineligible by title and abstract review and an additional 59 upon full-text review. We therefore included 128 observational studies (**Figure 1**).

We included 48 more studies in this update compared to our previous review. Of the total 3 532 258 pregnant women within the included studies (with sample sizes ranging from 701 to 267 834), 47 285 had placenta previa. The included studies encompassed all provinces of mainland China, with 30 studies conducted in East China, 26 in the South China, 22 in North China, 16 in Southwest China, 15 in Central China, 11 in Northeast China, and 9 in Northwest China. Ten studies were conducted after the implementation of the two-child policy (Table S1 in the **Online Supplementary Document**).

### Quality assessment

The median quality assessment rating was 5 (interquartile range = 5–7). Of the 128 studies that reported prevalence of placenta previa, 11 (8.6%) scored 9 points, 13 (10.2%) scored 8 points, 13 (10.2%) scored 7 points, 28 (21.9%) scored 6 points, 48 (37.5%) scored 5 points, and 15 (11.7%) scored 4 points (Table S2 in the **Online Supplementary Document**).

### Overall prevalence of placenta previa

The overall pooled prevalence of placenta previa was 1.44% (95% confidence interval (CI) = 1.32, 1.56). The prevalence of placenta previa increased significantly after the implementation of the two-child policy, from 1.25% (95% CI = 1.16, 1.34) in the prior period to 4.12% (95% CI = 3.33, 4.91) after the implementation (**Table 1**).

**Table 1:** Prevalence of placenta previa in mainland China and subgroup analysis

Variable	Number of surveys	Sample size	Placenta previa cases	Prevalence, per 100 000 population (95% CI)	*I* ^2^
Overall prevalence	128	3 532 258	47 285	1.44 (1.32, 1.56)	99.4
Two-child policy					
*Before*	127	2 287 221	24 458	1.25 (1.16, 1.34)	98.4
*After*	10	274 564	10 179	4.12 (3.33, 4.91)	99.1
Region					
*Northeast*	11	84 668	966	1.30 (0.94, 1.66)	96.5
*North*	22	1 188 342	7925	1.17 (1.02, 1.32)	99.1
*Northwest*	9	70 020	735	1.13 (0.73, 1.53)	97.5
*Central China*	15	222 649	2666	1.57 (1.25, 1.89)	98.5
*East*	30	884 321	13 199	1.18 (0.88, 1.47)	99.3
*South*	26	368 592	5373	1.46 (1.18, 1.73)	98.3
*Southwest*	16	298 951	10 408	2.34 (1.55, 3.13)	99.4
Survey year					
*1960–69*	1	10 919	220	2.01 (1.75, 2.28)	-
*1970–79*	4	197 158	1058	1.17 (0.53, 1.80)	99.3
*1980–89*	18	305 270	2610	0.91 (0.74, 1.07)	96.5
*1990–99*	18	187 323	1932	1.23 (1.00, 1.47)	96.8
*2000–09*	42	348 137	3765	1.17 (1.05, 1.30)	92.3
*2010–15*	52	2 208 887	27 521	1.47 (1.29, 1.66)	99.6
*2016–present*	10	274 564	10 179	4.12 (3.33, 4.91)	99.1
Age					
*<25*	5	41 404	463	1.23 (0.79, 1.67)	93.9
*25–30*	56	1 418 991	13954	1.27 (1.13, 1.41)	98.8
*≥30*	46	884 899	15248	1.68 (1.42, 1.94)	99.3
Quality score					
*0–5*	63	595 216	6492	1.23 (1.10, 1.35)	96.6
*6–10*	65	2 937 042	40 793	1.62 (1.44, 1.79)	99.7
Hospital level					
*Tertiary*	50	2 665 787	35 334	1.66 (1.46, 1.85)	99.7
*Secondary*	50	639 762	9359	1.36 (1.15, 1.57)	98.7
*Primary*	28	226 709	2592	1.22 (1.06, 1.39)	93.1

CI – confidence interval

### Subgroup analysis

**Figure 2:**
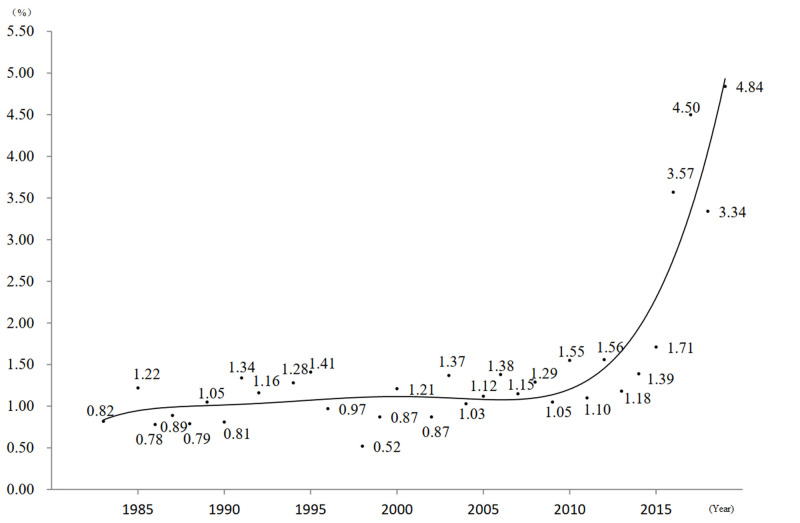
Trends in the prevalence of placenta previa.

At the regional level, the prevalence of placenta previa was the highest in Southwestern China (2.34%), followed by Central China (1.57%), South China (1.46%), Northeast China (1.30%), East China (1.18%), North China (1.17%), and Northwest China (1.13%). In the province-level analysis, we observed the highest prevalence in Hainan (2.90%), and the lowest in Xinjiang (0.43%) (Figure S1 and Table S3 in the **Online Supplementary Document**). In the subgroup analysis by year, the prevalence of placenta previa fluctuated slightly around 1.20% prior to 2015, except for 1993 (2.99%). Afterwards, the prevalence increased to 3.57% in 2016, 4.50% in 2017, 3.34% in 2018, and 4.84% in 2019 (**Figure 2**). We also observed that the prevalence of placenta previa increased with maternal age, from 1.23% at <25 years of age, to 1.27% at 25–30 years of age, and lastly to 1.68% at >30 years. At the hospital level, we observed the prevalence of placenta previa was 1.66% in tertiary hospitals, 1.36% in secondary hospitals, and 1.22% in primary hospitals. By quality score, the prevalence of placenta previa was significantly higher in the 6–10 scores group (1.62%) than in the 0–5 scores group (1.23%) (**Table 1**).

### Meta-regression analysis

Univariable meta-regression analyses showed that the implementation of the two-child policy and survey year were potential sources of heterogeneity. Multivariable analyses confirmed these findings and further showed that the two-child policy has the greatest influence on the result (**Table 2**).

**Table 2:** Meta-regression analysis on the included studies

	Univariable meta-regression			Multivariable meta-regression		
**Variables**	**Coefficient (95% CI)**	***t***	***P*-value**	**Coefficient (95% CI)**	***t***	***P*-value**
Two-child policy	0.0280 (0.0213, 0.0346)	8.37	0.0001	0.0292 (0.0178, 0.0406)	5.09	0.0001
Region	0.0008 (−0.0003, 0.0020)	1.37	0.1750	0.0006 (−0.0007, 0.0019)	0.95	0.3450
Survey year	0.0034 (0.0018, 0.0049)	4.34	0.0001	0.0025 (0.0002, 0.0049)	2.17	0.0330
Maternal age	0.0035 (−0.0008, 0.0078)	1.61	0.1110	−0.0003 (−0.0045, 0.0038)	−0.15	0.8780
Quality score	0.0031 (−0.0013, 0.0076)	1.39	0.1660	-0.0009 (−0.0057, 0.0040)	−0.36	0.7180
Hospital level	0.0023 (−0.0007, 0.0052)	1.53	0.1290	0.0008 (−0.0023, 0.0038)	0.49	0.6250

CI – confidence interval

## DISCUSSION

In this updated systematic review and meta-analysis based on pooled results from 128 studies involving 3 532 258 participants, we found the prevalence of placenta previa in mainland China to be 1.44%. We also observed a significant difference in the prevalence of placenta previa in the period before compared to the period after the implementation of the two-child policy (1.25% vs 4.12%), confirming our hypothesis that it increased the risk of placenta previa.

This is our second, updated meta-analysis assessing the prevalence of placenta previa in pregnant women in mainland China. In the previous review, we included 80 studies published before 2015, meaning they only included the period before the two-child policy [4]. Therefore, we were unable to assess the impact of the birth policy changes on placenta previa at that time.

While our main findings are consistent with our previous meta-analysis, meaning we again observed that the prevalence of placenta previa significantly varied across mainland China, this update included a higher number of studies and enabled us to conduct a separate analysis for the period after the two-child policy was implemented. Here we found that, in contrast to the minor fluctuations observed in the previous study, the prevalence of placenta previa increased sharply in the post-implementation period.

Since the introduction of the new two-child policy, the rates of advanced maternal age, multiparous women, history of caesarean section, and pregnancy complications increased in mainland China, leading to an increase in the rate of adverse pregnancy outcomes [10,16,21,38], leading to an increase in adverse pregnancy outcomes. With the further broadening of China’s childbearing policy, pregnancy complications – including placenta previa – will likely continue to rise, necessitating the attention of governmental institutions, including through early planning and prevention, increasing the number of obstetricians, and the establishment of high-standard regional maternity treatment centres.

Our analysis of geographical distribution showed that the prevalence has increased in all regions after the implementation of the two-child policy, with some distinct differences between regions. However, at this level, there has been no significant change in the relative prevalence of placenta previa compared to the period before the implementation of the policy. Specifically, we again observed that Hainan and Liaoning had the highest and lowest prevalence of placenta previa, respectively, compared to the other areas. These significant regional variations in placenta previa rates and trends may be related to the differences in geography, economy, living conditions, and other factors like patient preferences and health care systems. Simultaneously, the implementation of the two-child policy has also increased the occurrence of placenta previa through the impact of risk factors.

Like in other meta-analyses of the prevalence of placenta previa [3,28,29], we observed high heterogeneity between studies, which was not explained by our subgroup analyses of geographical study regions, survey years, quality scores, maternal age, and hospital levels. We propose that other factors, such as maternal behaviours, lifestyle habits, alcohol or coffee consumption, sociodemographic characteristics, and mental and physical inactivity may have influenced this heterogeneity. However, due to limited data on these factors, we were unable to carry out further analyses. We advise that our results are interpreted through the 95% CIs rather than the pooled result due to the high level of heterogeneity.

We also performed univariable and multivariable meta-regression analyses to further explore the size and the source of this heterogeneity. Our findings suggest that the survey year and the implementation of the two-child policy were significantly associated with the prevalence of placenta previa. Notably, the studies in our analysis were conducted between 1965 and 2023. During this nearly 60-year period, maternal behaviours, lifestyle habits, and sociodemographic characteristics of the participants have changed greatly, as did health care and reporting practices, as well as the criteria and methods for diagnosing of placenta previa.

Moreover, previous evidence indicated that the rates of advanced maternal age, women with prior caesarean sections and uterine scars, assisted reproductive technology interventions, and multiparous women were significantly increased after the implementation of the two-child policy [17,18]. The risk of placenta previa is likely significantly higher in women who experience these conditions or interventions, which will likely lead to a hike in the incidence of placenta previa in this population. This partly explains why the meta-regression analyses suggested that the two-child policy had the highest impact on the heterogeneity.

Placenta previa is a complex clinical and public health issue that both impacts the health of pregnant women and their offspring and contributes to a significant increase in the national disease burden [39–41]. The main contribution of our findings is a detailed representation of the currently alarming state of placenta previa, which could inform and alert decision-makers and practitioners in the health care sector in China – especially as the increase in the number of pregnant women at high risk of placenta previa will further exacerbate these challenges. Meanwhile, individuals, families, communities, and hospitals should also strive towards a multisectoral collaboration to ensure the safety of pregnant women with placenta previa.

The main strengths of this meta-analysis are its relatively large sample size and its novel estimates of the prevalence of placenta previa in pregnant women in mainland China after the implementation of the two-child policy. Of course, our findings must be interpreted in the context of the limitations of our study. Specifically, we observed high between-study heterogeneity in the analysis of placenta previa prevalence due to differences in the study years and regions. Moreover, as newer studies are yet to be published, we have only included 10 articles after the two-child policy, which may have affected the stability of our results, especially for the periods after this policy was implemented. Future studies could provide more data to address this gap. Moreover, this study only focussed on the change in the prevalence of placenta previa after two-child policy was implemented. We leave other specific clinical and public health issues for future studies, such as longitudinal studies assessing the long-term impact of the two-child policy on maternal health outcomes.

## CONCLUSIONS

The prevalence of placenta previa increased significantly from the one-child policy period to the two-child policy period among pregnant women in mainland China, with distinct variations across regions. This necessitates the attention of health officials and timely adjustment of resource allocation policies. Our findings provide a stronger basis for understanding the impact of policy changes on placenta previa prevalence in mainland China. Accurate early prediction models are needed to improve individualised care for affected women, while an early maternal referral system could help them access immediate care and thereby ensure their safety, especially in lower-level hospitals.

## Additional material


Online Supplementary Document.
