# Supplementary material for: Prevalence of placenta previa among deliveries: An update systematic review and meta-analysis after the introduction of the two-child policy in Mainland China
Source: J Glob Health. 2024 Jun 14;14:04108. doi: 10.7189/jogh.14.04108 (PMC11170231; doi:10.7189/jogh.14.04108)
Supplement: Online Supplementary Document. [file jogh-14-04108-s001.pdf]

Supplementary files

Supplementary file 1. The full search strategy. (P2)

Supplementary Table 1. Characteristics and quality scores of the included studies. (P3-7)
Supplementary Table 2. The detailed quality scores of the included studies. (P8-12)

Supplementary table 3. The prevalence of placenta previa in each province. (P18)

Supplementary figure 1. The provincial distribution of the prevalence of placenta previa on map. (P19)

Supplementary file 1. The full search strategy

(placenta OR “placenta previa” OR “placenta praevia” OR "placenta previa"[Mesh] OR PP) AND (incidence OR prevalence OR "Incidence"[MESH] OR "Prevalence"[MESH]) AND (China OR Chinese OR China[Affiliation] OR Chinese[Affiliation] OR China[Title/Abstract])

Supplementary Table 1. Characteristics and quality scores of the included studies.

Reference	Author/Year	Province	Enrolment Period	Sample size	Placenta previa size	Hospital Level	Quality Score
[1]	He et al./2022	Shanghai	201301-201810	16198	719	Tertiary	9
[2]	Zhang et al./2022	National	201001-201712	215254	676	Tertiary	8
[3]	Li et al./2021	National	201610-201709	99253	2518	Tertiary	8
[4]	Rao et al./2021	Guangdong	201201-201704	58062	726	Secondary	9
[5]	Huang et al./2021	Jiangsu	201010-201712	29520	493	Tertiary	7
[6]	Xu et al./2020	Shanxi	201304-201605	151333	2128	Tertiary	8
[7]	Li et al./2020	Hebei	201301-201712	267834	1221	Tertiary	9
[8]	Li et al./2020	Yunnan	201601-201812	9515	329	Tertiary	5
[9]	Wang et al./2020	Chongqing	201401-201812	89769	3319	Secondary	8
[10]	Duan et al./2020	Hunan	201501-201808	4196	417	Tertiary	6
[11]	Zhang et al./2020	National	201101-201212	108059	1330	Tertiary	9
[12]	Li et al./2019	Shaanxi	201501-201810	3326	84	Tertiary	8
[13]	Jiang et al./2019	Jiangsu	201210-201712	24192	404	Tertiary	8
[14]	Zhang et al./2018	Beijing	201501-201703	10998	291	Tertiary	8
[15]	Jiang et al./2018	Shanghai	201111-201610	74444	678	Tertiary	8
[16]	Chen et al./2018	Chongqing	201401-201412	4827	146	Tertiary	7
[17]	Liu et al./2017	Jiangsu	200901-201508	28606	181	Tertiary	5
[18]	Yu et al./2016	Beijing	200801-201412	28837	316	Tertiary	9
[19]	Lv et al./2016	Sichuan	200501-201406	79304	3840	Tertiary	8
[20]	Wu et al./2015	Guangdong	201201-201306	9657	143	Secondary	5
[21]	Di et al./2015	Xinjiang	201307-201407	4972	5	Secondary	5
[22]	Luo et al./2015	National	201001-201112	111767	1367	Tertiary	9
[23]	Zhang et al./2015	Henan	201003-201305	9875	120	Secondary	6
[24]	Xi et al./2015	Inner Mongolia	201201-201312	2203	20	Primary	4
[25]	Yuan et al./2015	Jilin	201002-201410	701	68	Secondary	5
[26]	Chen et al./2015	Guangdong	200801-201401	14547	206	Primary	6
[27]	Ji et al./2015	Inner Mongolia	201001-201412	3636	112	Secondary	5

[28]	Cao et al./2015	Inner Mongolia	201003-201505	7920	120	Secondary	5
[29]	Yang et al./2014	Guizhou	200901-201401	7186	83	Primary	6
[30]	Fu et al./2014	Beijing	200901-201206	9086	222	Tertiary	8
[31]	Yu et al./2014	Zhejiang	201001-201307	4565	42	Primary	7
[32]	Li et al./2014	National	201101-201113	108049	1304	Tertiary	9
[33]	Li et al./2014	Inner Mongolia	201101-201306	16578	252	Tertiary	6
[34]	Zhu et al./2014	Jilin	200006-201010	5400	94	Primary	4
[35]	Yu et al./2014	Guangdong	200912-201212	3960	60	Primary	7
[36]	Yang et al./2014	Tibet	200801-201212	5986	61	Secondary	4
[37]	Li et al./2013	Gansu	200805-201201	10576	198	Tertiary	7
[38]	Feng et al./2013	Henan	200901-201101	2380	26	Primary	5
[39]	Zhao et al./2013	Shanxi	201001-201206	9056	102	Secondary	6
[40]	Li et al./2013	Guangdong	201101-201112	9385	117	Tertiary	8
[41]	Cheng et al./2013	Guizhou	200802-201302	986	14	Secondary	6
[42]	Chen et al./2013	Sichuan	200901-201012	34014	1432	Tertiary	8
[43]	Shao et al./2013	Anhui	200801-201112	8466	40	Secondary	5
[44]	Bai et al./2013	Henan	200601-201010	13464	219	Primary	6
[45]	Guo et al./2012	Guangdong	200802-201111	4010	62	Primary	7
[46]	Yan et al./2012	Jiangsu	200801-201112	9825	55	Secondary	6
[47]	Han et al./2012	Guangdong	200501-201205	4892	63	Primary	7
[48]	Zhang et al./2012	Shaanxi	200901-201112	3333	50	Tertiary	6
[49]	Liu et al./2012	Jilin	200605-200910	3426	35	Secondary	4
[50]	Chen et al./2012	Guangdong	200802-201111	3865	61	Primary	7
[51]	Zhou et al./2012	Guangxi	200801-201112	3165	58	Primary	7
[52]	Li et al./2012	Sichuan	200710-201109	19218	183	Primary	7
[53]	Liu et al./2012	Sichuan	200104-201108	10157	221	Secondary	6
[54]	Liu et al./2011	Hebei	200201-201012	3336	48	Primary	5
[55]	Du et al./2011	Tianjin	200001-201007	16330	110	Primary	6
[56]	Wang et al./2011	Inner Mongolia	200601-200912	5326	54	Secondary	5

[57]	Zhang et al./2011	Hainan	200001-200912	5568	46	Tertiary	5
[58]	Liu et al./2011	Hunan	200806-201105	24213	371	Tertiary	6
[59]	Wei et al./2010	Shaanxi	200501-200801	4810	47	Secondary	5
[60]	Sun et al./2010	Anhui	200501-200901	6040	90	Secondary	7
[61]	Wu et al./2010	Fujian	200401-200812	18741	225	Secondary	6
[62]	Jing et al./2010	Sichuan	200001-200812	9850	145	Primary	6
[63]	Zhang et al./2010	Ningxia	200401-200905	3774	40	Secondary	5
[64]	Han et al./2009	Tianjin	200001-200712	20128	104	Primary	6
[65]	Zhao et al./2009	Jilin	198901-200812	7026	52	Primary	5
[66]	Liao et al./2009	Jiangxi	199801-200801	23801	145	Secondary	4
[67]	Sun et al./2009	Jiangsu	200101-200412	10834	82	Secondary	5
[68]	Zheng et al./2008	Hunan	200006-200706	12834	86	Tertiary	7
[69]	Zhu et al./2008	Jiangsu	200501-200712	6530	68	Secondary	5
[70]	Tang et al./2008	Guangdong	200201-200801	8060	92	Primary	5
[71]	Wang et al./2008	Shanghai	200201-200806	36669	427	Secondary	8
[72]	Sun et al./2008	Beijing	200201-200701	11320	106	Secondary	5
[73]	Xiao et al./2007	Hunan	199901-200612	4628	72	Secondary	7
[74]	Liu et al./2007	Chongqing	200406-200606	1656	26	Secondary	5
[75]	Kong et al./2007	Guangdong	200601-200612	1496	23	Tertiary	5
[76]	Ye et al./2007	Anhui	200401-200512	8275	106	Secondary	5
[77]	Wei et al./2007	Guangdong	200001-200701	3016	46	Primary	5
[78]	Wu et al./2006	Guangdong	200201-200512	6140	143	Secondary	6
[79]	Hong et al./2006	Guangdong	199601-200401	16320	126	Secondary	6
[80]	Li et al./2006	Chongqing	200204-200504	2182	36	Secondary	5
[81]	Chen et al./2005	Anhui	200101-200412	4337	39	Secondary	5
[82]	Liu et al./2005	Jiangxi	199601-200312	5635	58	Primary	5
[83]	Liu et al./2004	Hebei	200101-200212	4121	43	Tertiary	4
[84]	Jiang et al./2003	Liaoning	199301-200112	8357	26	Secondary	5
[85]	Cheng et al./2003	National	199808-200102	14071	122	Tertiary	9

[86]	Liu et al./2002	Tianjin	199101-200012	13982	129	Secondary	4
[87]	Guo et al./2002	Hebei	200001-200012	1557	40	Secondary	5
[88]	Zhao et al./2002	Jilin	200001-200102	1832	20	Primary	5
[89]	Zhang et al./2001	Hubei	199101-200012	6782	219	Secondary	5
[90]	Chen et al./2001	Anhui	199206-200005	9018	95	Secondary	5
[91]	Cui et al./2001	Jilin	199001-199912	12146	78	Tertiary	5
[92]	Feng et al./1999	Shandong	198801-199701	25640	258	Secondary	5
[93]	Wang et al./1999	Hubei	199101-199805	33758	168	Tertiary	4
[94]	Xie et al./1999	Zhejiang	198501-199412	7443	68	Primary	4
[95]	He et al./1999	Zhejiang	197601-199808	26489	182	Primary	6
[96]	Yu et al./1998	Jiangsu	199201-199601	3860	56	Primary	5
[97]	Li et al./1998	Guangxi	198401-199412	28093	319	Secondary	6
[98]	Hao et al./1998	Shanxi	198201-199301	16746	173	Tertiary	5
[99]	Sheng et al./1998	Heilongjiang	198701-199601	4212	80	Secondary	4
[100]	Lei et al./1997	Qinghai	197601-199604	16667	110	Tertiary	5
[101]	Zhang et al./1997	Shanghai	199208-199601	2910	32	Tertiary	6
[102]	Li et al./1997	Hainan	199206-199506	5482	274	Secondary	5
[103]	Ye et al./1997	Zhejiang	198101-199412	18109	76	Secondary	5
[104]	Wang et al./1996	Henan	198501-199402	11215	84	Tertiary	6
[105]	Li et al./1995	Hubei	198001-199001	18945	360	Primary	5
[106]	Tang et al./1995	Guangxi	199001-199401	2854	46	Primary	6
[107]	Wu et al./1994	Guangdong	198301-199101	14052	137	Secondary	4
[108]	Cao et al./1994	Guangdong	199101-199201	4436	36	Secondary	5
[109]	Wang et al./1994	Beijing	196001-198912	139271	332	Tertiary	6
[110]	Kan et al./1994	Guangdong	198707-199012	3050	28	Primary	5
[111]	Li et al./1994	Guangdong	198306-199206	37012	128	Secondary	6
[112]	Chen et al./1993	Jiangsu	198101-199012	16336	184	Tertiary	6
[113]	Zhang et al./1993	Jiangsu	198701-199012	12280	136	Secondary	6
[114]	Mo et al./1993	Hunan	198101-199101	14314	128	Secondary	5

[115]	Li et al./1993	Jiangsu	198701-199012	11045	104	Secondary	4
[116]	Li et al./1992	Liaoning	199201-199212	14131	109	Secondary	4
[117]	Peng et al./1992	Shaanxi	198101-199012	11500	116	Tertiary	5
[118]	Zhang et al./1992	Hubei	198101-199012	35247	208	Tertiary	5
[119]	Tang et al./1991	Shandong	198704-199004	7383	92	Secondary	5
[120]	Wu et al./1990	Jiangsu	198001-198908	10417	67	Tertiary	4
[121]	Jiao et al./1989	Yunnan	198101-198512	7585	62	Tertiary	5
[122]	Zhang et al./1988	Henan	196601-198307	25257	162	Tertiary	6
[123]	Wang et al./1982	Guizhou	197101-198012	7030	161	Tertiary	5
[124]	Cai et al./1980	Guangdong	196401-197712	25600	403	Tertiary	4
[125]	Guo et al./1965	Heilongjiang	195601-196403	10919	220	Tertiary	5
[126]	Zhu et al./2024	Zhejiang	201001-202112	280203	7942	Tertiary	9
[127]	Tian et al./2023	Hebei	201301-202112	413892	1715	Tertiary	9
[128]	Fan et al./2023	Guangdong	201401-201906	68301	1713	Tertiary	9

Supplementary Table 2. The detailed quality scores of the included studies.

Reference	Author/Year	Selection			Performance bias		Quality Score
		Sample population	Sample size	Participation rate	Outcome assessment	Analytical methods to control for bias	
[1]	He et al./2022	2	2	2	2	1	9
[2]	Zhang et al./2022	2	2	1	2	1	8
[3]	Li et al./2021	2	2	1	2	1	8
[4]	Rao et al./2021	2	2	2	2	1	9
[5]	Huang et al./2021	2	2	1	1	1	7
[6]	Xu et al./2020	2	2	1	2	1	8
[7]	Li et al./2020	2	2	1	2	2	9
[8]	Li et al./2020	1	1	1	1	1	5
[9]	Wang et al./2020	2	2	1	2	1	8
[10]	Duan et al./2020	2	1	1	1	1	6
[11]	Zhang et al./2020	2	2	1	2	2	9
[12]	Li et al./2019	2	2	1	2	1	8
[13]	Jiang et al./2019	2	1	2	2	1	8
[14]	Zhang et al./2018	2	2	1	2	1	8
[15]	Jiang et al./2018	2	2	1	2	1	8
[16]	Chen et al./2018	2	1	1	2	1	7
[17]	Liu et al./2017	1	1	1	1	1	5
[18]	Yu et al./2016	2	2	1	2	2	9

[19]	Lv et al./2016	2	2	1	2	1	8
[20]	Wu et al./2015	1	1	1	1	1	5
[21]	Di et al./2015	1	1	1	1	1	5
[22]	Luo et al./2015	2	2	1	2	2	9
[23]	Zhang et al./2015	2	1	1	1	1	6
[24]	Xi et al./2015	1	1	0	1	1	4
[25]	Yuan et al./2015	1	1	1	1	1	5
[26]	Chen et al./2015	1	2	1	1	1	6
[27]	Ji et al./2015	1	1	1	1	1	5
[28]	Cao et al./2015	1	1	1	1	1	5
[29]	Yang et al./2014	2	1	1	1	1	6
[30]	Fu et al./2014	2	2	1	2	1	8
[31]	Yu et al./2014	2	2	1	1	1	7
[32]	Li et al./2014	2	2	1	2	2	9
[33]	Li et al./2014	2	1	1	1	1	6
[34]	Zhu et al./2014	1	1	0	1	1	4
[35]	Yu et al./2014	2	2	1	1	1	7
[36]	Yang et al./2014	1	1	0	1	1	4
[37]	Li et al./2013	2	1	1	2	1	7
[38]	Feng et al./2013	1	1	1	1	1	5
[39]	Zhao et al./2013	2	1	1	1	1	6
[40]	Li et al./2013	2	2	1	2	1	8
[41]	Cheng et al./2013	2	1	1	1	1	6
[42]	Chen et al./2013	2	2	2	1	1	8
[43]	Shao et al./2013	1	1	1	1	1	5
[44]	Bai et al./2013	2	1	1	1	1	6
[45]	Guo et al./2012	2	2	1	1	1	7
[46]	Yan et al./2012	2	1	1	1	1	6
[47]	Han et al./2012	2	1	2	1	1	7

[48]	Zhang et al./2012	2	1	1	1	1	6
[49]	Liu et al./2012	1	1	0	1	1	4
[50]	Chen et al./2012	2	2	1	1	1	7
[51]	Zhou et al./2012	2	2	1	1	1	7
[52]	Li et al./2012	2	2	1	1	1	7
[53]	Liu et al./2012	2	1	1	1	1	6
[54]	Liu et al./2011	1	1	1	1	1	5
[55]	Du et al./2011	2	2	1	1	1	6
[56]	Wang et al./2011	1	1	1	1	1	5
[57]	Zhang et al./2011	1	1	1	1	1	5
[58]	Liu et al./2011	2	1	1	1	1	6
[59]	Wei et al./2010	1	1	1	1	1	5
[60]	Sun et al./2010	2	2	1	1	1	7
[61]	Wu et al./2010	2	1	1	1	1	6
[62]	Jing et al./2010	1	2	1	1	1	6
[63]	Zhang et al./2010	1	1	1	1	1	5
[64]	Han et al./2009	2	1	1	1	1	6
[65]	Zhao et al./2009	1	1	1	1	1	5
[66]	Liao et al./2009	1	1	0	1	1	4
[67]	Sun et al./2009	1	1	1	1	1	5
[68]	Zheng et al./2008	2	2	1	1	1	7
[69]	Zhu et al./2008	1	1	1	1	1	5
[70]	Tang et al./2008	1	1	1	1	1	5
[71]	Wang et al./2008	2	2	1	2	1	8
[72]	Sun et al./2008	1	1	1	1	1	5
[73]	Xiao et al./2007	2	2	1	1	1	7
[74]	Liu et al./2007	1	1	1	1	1	5
[75]	Kong et al./2007	1	1	1	1	1	5
[76]	Ye et al./2007	1	1	1	1	1	5

[77]	Wei et al./2007	1	1	1	1	1	5
[78]	Wu et al./2006	2	1	1	1	1	6
[79]	Hong et al./2006	2	1	1	1	1	6
[80]	Li et al./2006	1	1	1	1	1	5
[81]	Chen et al./2005	1	1	1	1	1	5
[82]	Liu et al./2005	1	1	1	1	1	5
[83]	Liu et al./2004	1	1	0	1	1	4
[84]	Jiang et al./2003	1	1	1	1	1	5
[85]	Cheng et al./2003	2	2	2	2	1	9
[86]	Liu et al./2002	1	1	0	1	1	4
[87]	Guo et al./2002	1	1	1	1	1	5
[88]	Zhao et al./2002	1	1	1	1	1	5
[89]	Zhang et al./2001	1	1	1	1	1	5
[90]	Chen et al./2001	1	1	1	1	1	5
[91]	Cui et al./2001	1	1	1	1	1	5
[92]	Feng et al./1999	1	1	1	1	1	5
[93]	Wang et al./1999	1	1	0	1	1	4
[94]	Xie et al./1999	1	1	0	1	1	4
[95]	He et al./1999	2	1	1	2	1	6
[96]	Yu et al./1998	1	1	1	1	1	5
[97]	Li et al./1998	2	2	1	1	1	6
[98]	Hao et al./1998	1	1	1	1	1	5
[99]	Sheng et al./1998	1	1	0	1	1	4
[100]	Lei et al./1997	1	1	1	1	1	5
[101]	Zhang et al./1997	2	2	1	1	1	6
[102]	Li et al./1997	1	1	1	1	1	5
[103]	Ye et al./1997	1	1	1	1	1	5
[104]	Wang et al./1996	2	2	1	1	1	6
[105]	Li et al./1995	1	1	1	1	1	5

[106]	Tang et al./1995	2	2	1	1	1	6
[107]	Wu et al./1994	1	1	1	0	1	4
[108]	Cao et al./1994	1	1	1	1	1	5
[109]	Wang et al./1994	1	2	1	2	1	6
[110]	Kan et al./1994	1	1	1	1	1	5
[111]	Li et al./1994	2	1	1	1	1	6
[112]	Chen et al./1993	2	1	1	1	1	6
[113]	Zhang et al./1993	2	1	1	1	1	6
[114]	Mo et al./1993	1	1	1	1	1	5
[115]	Li et al./1993	1	1	0	1	1	4
[116]	Li et al./1992	1	1	1	0	1	4
[117]	Peng et al./1992	1	1	1	1	1	5
[118]	Zhang et al./1992	1	1	1	1	1	5
[119]	Tang et al./1991	1	1	1	1	1	5
[120]	Wu et al./1990	1	1	0	1	1	4
[121]	Jiao et al./1989	1	1	1	1	1	5
[122]	Zhang et al./1988	2	2	1	1	1	6
[123]	Wang et al./1982	1	1	1	1	1	5
[124]	Cai et al./1980	1	1	1	0	1	4
[125]	Guo et al./1965	1	1	1	1	1	5
[126]	Zhu et al./2024	2	2	2	2	1	9
[127]	Tian et al./2023	2	2	2	2	1	9
[128]	Fan et al./2023	2	2	2	2	1	9

References

1. He M, Sun X, Wang C, Sui Y. Analysis of the risk of complications during pregnancy in pregnant women with assisted reproductive technology: a retrospective study using registry linkage from 2013 to 2018 in Shanghai, China. BMC Pregnancy Childbirth. 2022;22:526.
2. Zhang YJ, Zhu Y, Zhu L, Lu CQ, Chen C, Yuan L. Prevalence of preterm birth and risk factors associated with it at different gestational ages: A multicenter

retrospective survey in China. Saudi Med J. 2022;43:599-609.
3. Li S, Gao J, Liu J, Hu J, Chen X, He J, *et al.* Incidence and Risk Factors of Postpartum Hemorrhage in China: A Multicenter Retrospective Study. Front Med (Lausanne). 2021;8:673500.
4. Rao J, Fan D, Zhou Z, Luo X, Ma H, Wan Y, *et al.* Maternal and Neonatal Outcomes of Placenta Previa with and without Coverage of a Uterine Scar: A Retrospective Cohort Study in a Tertiary Hospital. Int J Womens Health. 2021;13:671-681.
5. Huang S, Zuo Q, Wang T, Tang X, Ge Z, Lu H, *et al.* Maternal and neonatal outcomes of repeated antepartum bleeding in 493 placenta previa cases: a retrospective study. The Journal of Maternal-Fetal & Neonatal Medicine. 2021:1-6.
6. Xu C, Zhong W, Fu Q, Yi L, Deng Y, Cheng Z, *et al.* Differential effects of different delivery methods on progression to severe postpartum hemorrhage between Chinese nulliparous and multiparous women: a retrospective cohort study. BMC Pregnancy Childbirth. 2020;20:660.
7. Li SS, Guo GL, Yu C, Zhao S, Duan Y, Li JB, *et al.* Epidemiological characteristics and risk factors of placenta previa in Hebei Province. J Prac Med. 2020;36:249-253.
8. Li Y, Luo XM. Effects of different types of placenta previa on newborns. China Prac Med. 2020;15:73-75.
9. Wang Y, Li LJ, Liao YS. Analysis of obstetirc quality in Yuzhong District, Chongqing from 2014 to 2018. J Mod Med Health. 2020;36:2473-2476.
10. Duan WF, Zhang WS, Zhang JJ, Huang QR. Advanced maternal age and perinatal outcomes. Prog Obstet Gynecol. 2020;29:429-433.
11. Zhang XL, Liang H, Zhao HQ, Wu SW, Zhou QJ, Li XT. Optimized cutoff maternal age for adverse obstetrical outcomes: a multicenter retrospective cohort study in Urban China during 2011 to 2012. Chin Med J (Engl). 2020;133:269-276.
12. Li L, Zou AM, Shan L. Epidemiological investigation of placenta previa in Xi'an and its related risk factors and their influence on maternal and fetal outcomes. J Prev Med Chin PLA. 2019;37:106-109.
13. Jiang ZY, Sun LZ, Zhou X, Huang SY, Zuo Q, Ge ZP. Analysis of the relationship between prenatal bleeding and pregnancy outcome of placenta previa. Prac Gyn & Obs. 2019;35:214-220.
14. Zhang HX, Zhao YY, Wang YQ. Analysis of the Characteristics of Pregnancy and Delivery before and after Implementation of the Two-child Policy. Chin Med J (Engl). 2018;131:37-42.
15. Jing L, Wei G, Mengfan S, Yanyan H. Effect of site of placentation on pregnancy outcomes in patients with placenta previa. PLoS One. 2018;13:e0200252.
16. Chen JK, Zhu DW, Zheng YR, Yu LL, Yan YH, Zhang QH, *et al.* Distribution of Pregnancy Diseases and Pregnancy Outcomes in 4827 Pregnant Women. J Prac Obst Gyne. 2018;34:38-41.
17. Liu YY, You ZS, Ren XQ. Effect of placenta previa on pregnancy outcome in 181 cases. Chin J Clin Obstet Gynecol. 2017;18:73-74.
18. Yu L, Hu KJ, Yang HX. [A retrospective analysis on the pernicious placenta previa from 2008 to 2014]. Zhonghua Fu Chan Ke Za Zhi. 2016;51:169-173.
19. Lv B, Chen M, Liu XH. Risk factors of peripartum hysterectomy in placenta previa: a retrospective study of 3 840 cases. Chin J Obstet Gynecol. 2016;51:498-502.
20. Wu YM, Zhan MX, Wang Y. Analysis and nursing of placenta previa pregnancy outcome. China Medical Engineering. 2015;23:188-192.
21. Dili SB. Clinical analysis of 56 cases of placenta previa. World Latest Medicine Information. 2015;15:53-58.
22. Luo XL, Zhang WY. Obstetrical disease spectrum in China: an epidemiological study of 111,767 cases in 2011. Chin Med J (Engl). 2015;128:1137-1146.
23. Zhang MT. Analysis on pathogenic factors and pregnancy outcome of placenta previa. China Medicine and Pharmacy. 2015;5:77-79.
24. Xi QL. Clinical analysis of 20 cases of placenta previa. J Clin Med. 2015;2:1674.
25. Yuan LL. Clinical analysis of 68 cases of placenta previa. China Prac Med. 2015;10:268-269.
26. Chen LY, Li SY, Chen J, Lin L. Clinical analysis of placenta previa combined with placenta accreta. Prog Obstet Gynecol. 2015;24:123-125.
27. Ji XL, Chen SR, Shang YH. Clinical Analysis of 112 Patients With Placenta Previa. Inner Mongolia Med J. 2015;47:1050-1053.

28. Cao W. Analysis of influencing factors of placenta previa and its pregnancy outcome. World Latest Medicine Information. 2015;15:64-65.
29. Yang XM. Clinical analysis of 83 cases of placenta previa. Guide of China Medicine. 2014;12:121-123.
30. Fu CW, Liu YT, Yang JQ, Zhou L, Bian XM, Gao JS*, et al.* Placenta previa and postpartum hemorrhage: case analysis of 222 patients with placenta previa. J Reprod Med. 2014;23:224-228.
31. Yu LQ. The risk factors of placenta previa: a case-control study. Chin. J of PHM. 2014;30:292-293.
32. Li Q, Wang ZJ, Yu YH, Guo SQ, Guo XL, Xia YY*, et al.* Epidemiological survey on placenta previa in seven regions of China. Chinese Journal of Practical Gynecology and Obstetrics. 2014;30:786-790.
33. Li T, Yang XY, Liu AJ, Zhu XX. Clinical analysis of 252 cases of complete placenta previa. Inner Mongolia Med J. 2014;46:340-342.
34. Yang X, Yang MF, Liu XC. Clinical analysis of 94 cases of placenta previa. Chin Heal Care & Nutri. 2014;5:2440-2441.
35. Yu YT. Analysis of risk factors associated with placenta previa and its effect on pregnancy. China Prac Med. 2014;9:7-9.
36. Yang XY. Clinical analysis of 78 cases of placenta previa in Lasa, Tibet. Chin Heal Care & Nutri. 2014;7:4770.
37. Li FP, Tuo SM, Zhao YH. Analysis of placenta previa risk factors. Journal of Lanzhou University (Medical Sciences). 2013;39:22-24.
38. Feng WL. Clinical analysis of 26 placenta previa cases with cesarean section. Chinese Community Doctors. 2013;15:40.
39. Zhao ZP. Analysis of 102 cases of placenta previa. Shanxi Med J. 2013;42:311-312.
40. Li Q, Yu YH, Wang ZJ, Guo SQ, Xia YY, Guo XL*, et al.* The effect of placenta previa, implantation on maternal and neonatal outcomes. Prog Obstet Gynecol. 2013;22:738-740.
41. Cheng H. The relationship between placental adhesion, placenta previa, placenta implantation and artificial abortion. China & Foreign Medical Treatment. 2013:106-107.
42. Chen M, Zhang L, Wei Q, Fu X, Gao Q, Liu X. Peripartum hysterectomy between 2009 and 2010 in Sichuan, China. Int J Gynaecol Obstet. 2013;120:183-186.
43. Shao XY. Clinical analysis of 40 cases of Placenta previa. J Anhui Health Voca Tech College. 2013;12:41-42.
44. Bai JE. Etiology analysis and nursing experience of placenta previa. Journal of Henan Medical College for Staff and Workers. 2013;25:728-729.
45. Guo DT. Relation of placenta previa, placenta adhesion and placenta implantation with artificial abortion. China Modern Medicine. 2012;19:166-167.
46. Yan YH, Zhang YX. Clinical analysis of 55 cases of placenta previa. China Prac Med. 2012;7:91-92.
47. Han Y, Zhao CW. Study on influence of placenta previa morbidity factors and its effect on pregnancy outcome. China Modern Medicine. 2012;19:53-54.
48. Zhang HL, Li LC, Zhang J. Analysis of risk factors and prognosis of placenta previa. Chinese Journal of Aesthetic Medicine. 2012;21:56-57.
49. Liu ZH. The clinical analysis of 20 cases placenta previa combined with placenta accrete. Guide of China Medicine. 2012;10:363-364.
50. Chen HY. The association between placenta previa, placenta adhesion, placenta implantation and artificial abortion. Chinese and Foreign Medical Research. 2012;10:124-125.
51. Zhou YH, Huang YJ. The analysis between prenatal check condition and pregnancy outcomes in Pingguo, Guangxi. Guangxi Medical Journal 2012;34:1081-1084.
52. Li YL. The role of the pregnancy management of placenta praevia in the pregnancy outcome. China Modern Doctor. 2012;50:155-156.
53. Liu YL, Wu LH. Clinical analysis of 68 cases of placenta previa. Chin Heal Nutri. 2012;8:417.
54. Liu SL. Clinical analysis of 48 cases of placenta previa. Chin Manipula & Rehabi Med. 2011;5:255-256.
55. Du J. Clinical analysis of 110 cases of placenta previa. J Med Theor & Prac. 2011;24:813-814.
56. Wang J. Clinical analysis of 54 cases of placenta previa. J North China Coal Medical University. 2011;13:73-74.
57. Zhang LJ, Wei T, Huang C. The association between placenta previa and abortion: a 46 cases study. J Third Mil Med Univ. 2011;33:1425.
58. Liu YF. Clinical analysis of 35 cases of placenta previa combined with placenta accreta. China Modern Medicine. 2011;18:41-42.

59. Wei MX. Treatment of pregnancy complicated with placenta previa. The Medical Forum. 2010;14:118-119.
60. Sun LF. Clinical observation of expectant treatment for placenta previa. Chin J Prim Med Pharm. 2010;17:2075.
61. Wu XY, Ding HM. Pathogeny analysis of 225 cases of placenta previa. Medical Information. 2010:553-554.
62. Jing ZY. Clinical Analysis of 145 Cases of placenta praevia. Medical Information. 2010:755-756.
63. Zhang HY, Zhou XY. Clinical analysis of 40 cases of placenta previa. Ning Xia Med J. 2010;32:1220.
64. Han YX. Clinical analysis of 104 cases of placenta previa. Capital Medicine. 2009;10:37.
65. Zhao QC, Lu B. Clinical analysis of 52 cases of placenta previa. Chin J Ethnomed Ethnopha. 2009;18:119.
66. Liao MF, Hu AZ, Gong CY. Clinical analysis of 145 cases of placenta previa. J Clin Exper Med. 2009;8:122.
67. Rui S. Clinical analysis of 82 cases of placenta previa. Contemporary Medicine. 2009;15:38.
68. Zheng AJ. Affection of placenta previa on the perineonate Chinese Journal of Woman and Child Health Research. 2008;19:331-332.
69. Zhu S. Clinical analysis of 68 cases of placenta previa. Jouranl of Modern Medicine & Health. 2008;24:1163-1164.
70. Tang L, Li X. Clinical analysis of 11 cases of placenta implantation. Jouranl of Gannan Medical University. 2008;28:538-539.
71. Wang AY, Huang Y. The relationship between placenta prvia with obstetric risk factor: a report of 427 cases. Chongqing Medicine. 2008;37:2310-2312.
72. Sun LY. Analysis of 106 cases of placenta previa and 7 case of placenta accrete. Medical Journal of Chinese People's Health. 2008;20:1549-1550.
73. Xiao ZS, Xie JP. Relation of Placenta Previa, Placenta Adhesion and Placenta Implantation With Artificial Abortion. Practical Preventive Medicine. 2007;14:1831-1832.
74. Liu LF. Clinical analysis of 26 cases of placenta previa. Chongqing Medicine. 2007;36:1340-1341.
75. Kong X, Zhang JH, Li Q, Huang XX. Clinical analysis of 5 cases of placenta previa and implantation The Journal of Practical Medicine. 2007;23:3593-3594.
76. Ye JL, Liu HM. Clinical analysis of 106 gravidas with placenta praevia. J Anhui Health Voca Tech College. 2007;6:25-26.
77. Wei LH. Clinical analysis of 46 cases of placenta previa. CHINA MEDICAL HERALD. 2007;4:149-150.
78. Wu FM, Zhao LY, Gao LJ. Clinical analysis of 143 cases of placenta previa. CHINA MEDICAL HERALD. 2006;3:37-39.
79. Hong XQ. Clincal Analysis of 126 Cases of Placenta Previa and 3 Cases of Placenta Accrete. Jouranl of Tropical Medicine. 2005;6:444-445.
80. Li ZR. Clinical analysis of 36 cases of placenta previa. Chongqing Medicine. 2006;35:575-576.
81. Chen JT. Retrospective analysis of pregnant outcomes in 39 gravidas with placenta previa. Anhui Med Pharm J. 2005;9:610-612.
82. Liu YE. Clinical analysis of 58 cases of placenta previa. J Gannan Med Coll. 2005;25:82-83.
83. Liu ZY, Liang YJ, Gao W, Liang L, Tian Z. Clinical analysis of 43cases of placenta previa. J Hebei Med Coll Cont Edu. 2004;21:21.
84. Jiang M, Tao YJ. Analysis to parturition manier of 26 placenta previas. Jouranl of Dalian Medical Uinversity. 2003;25:200-201.
85. Cheng YM, Yuan W, Cai WD, Zhang WM, Wang TY, Wang Y*, et al.* Study on the occurence of cesarean section (CS) and factors related to CS in China. Chin J Epidemiol 2003;10:893-896.
86. Liu ZP. Clinical analysis of 129 cases of placenta previa. Chinese Jouranl of Urban and Rural Enterprise Hygiene. 2002:29-31.
87. Guo LN, Wang XY. Clinical analysis of 40cases of placenta previa. J Med Theor & Prac. 2002;15:438-439.
88. Zhao MQ, Cui SY, Dong HJ, Wang SX, Wang YJ. Clinical analysis of 20 cases of Placenta previa. Chin Matern Child Healt Care. 2002;17:636-637.
89. Zhang LF. Research into the Incurrence Rate and Prognosis of Placenta Praevia. J. of Wuhan Uni. of Sci. & Tech. (Natural Science Edition). 2001;24:425-426.
90. Chen XZ. Clinical analysis of 68 cases of placenta previa. Chin J Prim Med Phar. 2001;8:191.
91. Cui ZH, Zhang XW, Jin GH, Li SH. Clinical analysis of 78 cases of placenta previa. J Med Sci Yanbian Uni. 2001;24:229-230.
92. Feng FZ, Zhang LH, Fang SL. The effect of magnesium sulfate in treatment of placenta previa. The Journal of Medical Theory and Practice. 1999;12:97-98.

93. Wang JF. Nursing care of patients in placenta previa with postpartum hemorrhage Journal of Nursing Science. 1999;14:159.
94. Xie RH. The association between placenta previa and maternal, infant prognosis. Zhejiang Clinical Medical Journal. 1999;1:185-186.
95. He CL. Clinical analysis of 182 cases of placenta previa. Clinical Medicine. 1999;19:28-29.
96. Yu WP. The effect of expectant treatment in placenta previa. Medical Journal of Communications. 1998;12:201.
97. Li MY. Clinical analysis of 319 cases of placenta previa. Journal of Youjiang Medical College for National Minorities. 1998;20:54-55.
98. Hao XY, Wang XH. The effect of placenta previa in the mother and infant. JOURNAL OF DATONG MEDICAL COLLEGE. 1998;18:16-18.
99. Sheng XB, Wang HQ, Zhang ZQ. The risk factors of vaginal bleeding in late pregnancy. Chinese Primary Health Care. 1998;12:32.
100. Lei YX. Clinical analysis of 110 cases of placenta previa. Jouranl of Practical Medical Techniques. 1997;4:234-235.
101. Zhang GH, Zhang DL. Clinical analysis of 32 cases of placenta previa. J Shanghai Tiedao Univ (Med Sci). 1997;11:42-43.
102. Li DM. Clinical analysis of 274 cases of placenta previa. Jouranl of Beijing College of Acupuncture-Moxibustion & Orthopedics-Traumatology. 1997;4:38-40.
103. Ye LL. Clinical analysis of 47 cases of placenta previa. HENAN MEDICAL INFORMATION. 1997;5:30-31.
104. Wang AY, Wang CP, Ma N. Clinical analysis of 84 cases of placenta previa. CENTRAL PLAINS MEDICAL JOURNAL. 1996;23:11-12.
105. Li JR. The delivery mode in placenta previa. Wuhan Medicine Journal. 1995;19:173-174.
106. Tang FX. Clinical analysis of 46 cases of placenta previa. Journal of Guilin Medical College. 1995;8:410-412.
107. Wu WL. Analysis of placenta previa in 137 cases. Guangdong Medical Journal. 1994;15:99-101.
108. Cao Y. Clinical analysis of 36 cases of placenta previa. JOURNAL OF LANZHOU MEDICAL COLLEGE. 1994;20:271.
109. Wang TT, Liu WF, Cong KJ. The diagnosis treatment and prognosis of placenta previa. BEIJING MEDICAL JOURNAL. 1994;16:114-115.
110. Kan M, Wang YY. Clinical analysis of 28 cases of placenta previa. MEDICAL JOURNAL OF LIAONING. 1994;8:161.
111. Li LW. Clinical analysis of 128 cases of placenta previa. The Journal of Practical Medicine. 1994;10:723.
112. Chen XL, Shen YJ. Clinical analysis of 184 cases of placenta previa. Jouranl of Nanjing Railway Medical College. 1993;12:33-35.
113. Zhang XZ, Yang TR. Clinical analysis of 126 cases of placenta previa. Jiangsu Medical Journal. 1993;19:395.
114. Mo DY, Jia Q, Zhang FJ. Clinical analysis of 128 cases of placenta previa. Hunan Medical Journal. 1993;11:62-63.
115. Li P, Zhang ZX, Qian S. Clinical analysis of 104 cases of placenta previa. Railway Medical Journal. 1993;21:156-157.
116. Li XX. Diagnosis and treatment of late pregnancy bleeding. MEDICAL JOURNAL OF LIAONING. 1992;6:129-130.
117. Peng JJ, Huang F, Yang HG, Zhang XL. The prevalence rate of placenta previa in perinatal period. Chinese Journal of Birth Health & Heredity. 1992;68:68-71.
118. Zhang YF, Li WJ. A Clinical Analysis of 109 Cases of Postpartum Hemorrhage Induced by Placenta Previa. Journal of Tongji Medical University. 1992;21:335-337.
119. Tang NF, Song XX. The association between placenta previa and artificial abortion. JOURNAL OF JINING MEDICAL COLLEGE. 1991;14:46-48.
120. Wu XY. The treatment of placenta previa. Journal of Medical Postgraduates. 1990;3:128-129.
121. Jiao SY, Xiao H, Li M. Clinical analysis of 62 cases of placenta previa. Medical and Pharmacy of Yunnan. 1989:170-171.
122. Zhang SJ, Sun YZ, Zhu XQ. Clinical analysis of 162 cases of placenta previa. Chinese Journal of Practical Gynecology and Obstetrics. 1988;4:27.
123. Wang JY. Clinical analysis of 116 cases of placenta previa. Guizhou Medical Journal. 1982:39.
124. Cai SY. Placenta Praevia - Analysis of 403 Cases. ACTA ACADEMIAE MEDICINAE ZHONG SHAN. 1980;1:312-316.
125. Guo JD, Zhang JY, Han XY. Clinical analysis of 220 cases of placenta previa. Journal of Harbin Medical University. 1965:90-95.
126. Zhu H, Cai J, Liu H, Zhao Z, Chen Y, Wang P, et al. Trajectories tracking of maternal and neonatal health in eastern China from 2010 to 2021: A multicentre cross-sectional study. J Glob Health. 2024;14:04069.
127. Tian ML, Ma GJ, Du LY, Jin Y, Zhang C, Xiao YG, et al. The Effect of 2016 Chinese second-child policy and different maternal age on pregnancy outcomes in Hebei

Province, China. BMC Pregnancy Childbirth. 2023;23:267.
128. Fan D, Rao J, Zhang H, Lin D, Guo X, Liu Z. Blood Type and Outcomes in Pregnant Women with Placenta Previa. Oxid Med Cell Longev. 2023;2023:4725064.

Supplementary table 3. The prevalence of placenta previa in each province.

Province	Number of surveys	Sample size	Placenta previa cases	Prevalence (per 100) [95%CI]
Anhui	5	36136	370	1.03 [0.65, 1.41]
Beijing	5	199512	1267	1.46 [0.71, 2.21]
Chongqing	4	98434	3527	2.51 [1.36, 3.65]
Fujian	1	18741	225	1.20 [1.05, 1.36]
Gansu	1	10576	198	1.87 [1.61, 2.13]
Guangdong	21	326251	4685	1.33 [1.03, 1.64]
Guangxi	3	34112	423	1.49 [1.01, 1.96]
Guizhou	3	15202	258	1.63 [0.80, 2.46]
Hainan	2	11050	320	2.90 [1.19, 6.99]
Hebei	5	690740	3067	0.61 [0.49, 0.72]
Heilongjiang	2	15131	300	1.98 [1.76, 2.20]
Henan	5	62191	611	1.06 [0.69, 1.43]
Hubei	4	94732	955	1.51 [0.89, 2.13]
Hunan	5	60185	1074	2.70 [1.75, 3.66]
Inner Mongolia	5	35663	558	1.56 [1.09, 2.03]
Jiangsu	11	163445	1830	1.05 [0.79, 1.30]
Jiangxi	2	29436	203	0.80 [0.39, 1.21]
Jilin	6	30531	347	1.46 [0.91, 2.01]
Liaoning	2	22488	135	0.54 [0.09, 0.99]
Ningxia	1	3774	40	1.06 [0.73, 1.39]
Qinghai	1	16667	110	0.66 [0.54, 0.78]
Shaanxi	4	22969	297	1.45 [0.93, 1.97]
Shandong	2	33023	350	1.10 [0.87, 1.33]
Shanghai	4	130221	1856	1.89 [1.00, 2.79]
Shanxi	3	177135	2403	1.20 [0.93, 1.47]
Sichuan	6	162229	6171	2.88 [1.32, 4.43]
Tianjin	3	50440	343	0.70 [0.48, 0.92]
Tibet	1	5986	61	1.02 [0.77, 1.27]
Xinjiang	2	16034	90	0.43 [0.22, 1.09]
Yunnan	2	17100	391	2.13 [0.45, 4.72]
Zhejiang	5	336809	8310	1.16 [0.57, 2.37]

Supplementary figure 1. The provincial distribution of the prevalence of placenta previa on map.

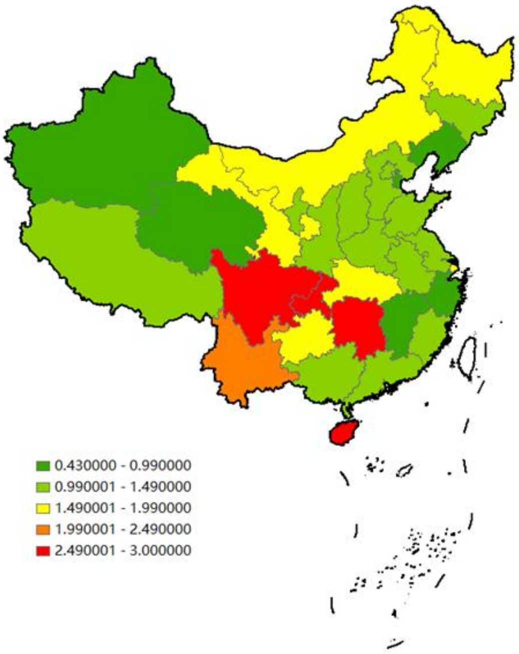

The highest prevalence of placenta previa was observed in Hainan (2.90%), and following in Sichuan (2.88%), Hunan (2.70%), Chongqing (2.51%), Yunnan (2.13%), Heilongjiang (1.98%), Shanghai (1.89%), Gansu (1.87%), Guizhou (1.63%), Inner Mongolia (1.56%), Hubei (1.51%), Guangxi (1.49%), Beijing (1.46%), Jilin (1.45%), Shaanxi (1.45%), Guangdong (1.33%), Fujian (1.20%), Shanxi (1.20%), Zhejiang (1.16%), Shandong (1.10%), Henan (1.06%), Ningxia (1.06%), Jiangsu (1.06%), Anhui (1.03%), Tibet (1.02%), Jiangxi (0.80%), Tianjin (0.70%), Qinghai (0.66%), Hebei (0.61%), Liaoning (0.54%), and the lowest prevalence was observed in Xinjiang (0.43%).
